# Smartphone-Acquired Anterior Segment Images for Deep Learning Prediction of Anterior Chamber Depth: A Proof-of-Concept Study

**DOI:** 10.3389/fmed.2022.912214

**Published:** 2022-06-23

**Authors:** Chaoxu Qian, Yixing Jiang, Zhi Da Soh, Ganesan Sakthi Selvam, Shuyuan Xiao, Yih-Chung Tham, Xinxing Xu, Yong Liu, Jun Li, Hua Zhong, Ching-Yu Cheng

**Affiliations:** ^1^Singapore Eye Research Institute, Singapore National Eye Centre, Singapore, Singapore; ^2^Department of Ophthalmology, The First Affiliated Hospital of Kunming Medical University, Kunming, China; ^3^Institute of High Performance Computing, Agency for Science, Technology and Research (A^*^Star), Singapore, Singapore; ^4^Department of Ophthalmology, Yong Loo Lin School of Medicine, National University of Singapore, Singapore, Singapore; ^5^Ophthalmology and Visual Sciences Academic Clinical Program (Eye ACP), Duke-NUS Medical School, Singapore, Singapore; ^6^Department of Ophthalmology, The Second People's Hospital of Yunnan Province, Kunming, China

**Keywords:** primary angle-closure glaucoma, glaucoma, anterior chamber depth, smartphone, deep learning

## Abstract

**Purpose:**

To develop a deep learning (DL) algorithm for predicting anterior chamber depth (ACD) from smartphone-acquired anterior segment photographs.

**Methods:**

For algorithm development, we included 4,157 eyes from 2,084 Chinese primary school students (aged 11–15 years) from Mojiang Myopia Progression Study (MMPS). All participants had with ACD measurement measured with Lenstar (LS 900) and anterior segment photographs acquired from a smartphone (iPhone Xs), which was mounted on slit lamp and under diffuses lighting. The anterior segment photographs were randomly selected by person into training (80%, no. of eyes = 3,326) and testing (20%, no. of eyes = 831) dataset. We excluded participants with intraocular surgery history or pronounced corneal haze. A convolutional neural network was developed to predict ACD based on these anterior segment photographs. To determine the accuracy of our algorithm, we measured the mean absolute error (MAE) and coefficient of determination (*R*^2^) were evaluated. Bland Altman plot was used to illustrate the agreement between DL-predicted and measured ACD values.

**Results:**

In the test set of 831 eyes, the mean measured ACD was 3.06 ± 0.25 mm, and the mean DL-predicted ACD was 3.10 ± 0.20 mm. The MAE was 0.16 ± 0.13 mm, and *R*^2^ was 0.40 between the predicted and measured ACD. The overall mean difference was −0.04 ± 0.20 mm, with 95% limits of agreement ranging between −0.43 and 0.34 mm. The generated saliency maps showed that the algorithm mainly utilized central corneal region (i.e., the site where ACD is clinically measured typically) in making its prediction, providing further plausibility to the algorithm's prediction.

**Conclusions:**

We developed a DL algorithm to estimate ACD based on smartphone-acquired anterior segment photographs. Upon further validation, our algorithm may be further refined for use as a ACD screening tool in rural localities where means of assessing ocular biometry is not readily available. This is particularly important in China where the risk of primary angle closure disease is high and often undetected.

## Introduction

Primary angle-closure glaucoma (PACG) is a significant cause of vision loss in Asia. It was estimated that the number of people aged 40–80 years with PACG worldwide was 23.36 million in 2020, of which Asia accounted for 76.8% of cases (1). Bilateral blindness affected 5.3 million people with PACG in 2020, the majority of whom were from Asian regions (2). Thus, screening for people with high risks of PACG is important to provide timely interventions, particularly in Asian countries (3).

Anterior chamber depth (ACD), the distance from corneal endothelium to the anterior crystalline lens capsule, is an important biometric dimension to assess the risk of angle closure development. A population-based study reported that ACD was a significant risk factor for angle closure amongst Mongolia and Chinese (4). Another population-based longitudinal study in China demonstrated that shallow ACD was independently associated with angle closure development over a 6-year period (5). Anterior chamber depth alone may provide a simple and effective way to distinguish eyes with angle closure from those with open angles (6), and has been suggested as a quick screening tool for detecting primary angle closure disease (PACD) (7, 8).

Currently, the methods used for ACD measurement include A-Scan ultrasound, slit-lamp biomicroscopy, non-contact partial coherence interferometry [e.g., IOLMaster (Carl Zeiss AG, Oberkochen, Germany), Lenstar (Lenstar LS 900®, Haag-Streit AG, Switzerland), Pentacam (Oculus System, Wetzlar, Germany)], and anterior segment optical coherence tomography (AS-OCT) (6, 9). However, the need for technical expertise, along with the cost and lack of portability, limit their usage in community screening (8, 9). The advent of artificial intelligence has made tremendous breakthroughs in ophthalmic imaging and shown great capabilities in disease diagnosis and screening (10). In recent times, Chen et al. developed a machine learning algorithm to predict ACD from images captured by a smartphone mounted with a portable slit lamp (*n* = 66) (11). In brief, the portable slit lamp was placed in front of the eye parallel to the cornea. The slit beam focused on the mid-peripheral iris surface, not too center nor too peripheral. Multiple images were captured in ~1 mm steps from nasal to temporal. Although their algorithm-predicted ACD showed moderate correlation with the measured ACD measurements, the need for manual maneuvering across the cornea with a 1 mm slit was subjective and time-consuming.

The availability of portable smartphones with cameras has become a tool for ophthalmologists in clinics (11–13). Using smartphones to take anterior segment photographs provide good reproducibility (12), and could provide clinicians with a simple and quick way to obtain anterior segment photographs for evaluation in rural or less-resourced areas.

In the present study, we aimed to develop and validate a DL algorithm for quantitative prediction of ACD from anterior segment photographs that were captured by a smartphone. This approach may provide clinicians with a mean to obtain ACD measurements in settings where biometers and advanced imaging tools are not readily available.

## Methods

### Study Population

The Mojiang Myopia Progression Study (MMPS) is a longitudinal school-based study that evaluates the onset and progression of myopia in school-aged children in rural China. Details of the methodology have been described previously (14–17). In brief, this study was conducted in Mojiang, a small country in Yunnan Province in the Southwestern part of China. A total of 2,432 elementary students (response rate 90.2%) and 2,346 middle school students (response rate 93.5%) were enrolled in the MMPS. The baseline examinations were conducted in 2016 and the MMPS participants were followed annually. The data used for the present study were from 2,195 elementary students participated in the 5-year follow up visit in 2020 (response rate 99.1%).

All study procedures were performed in accordance with the tenets of the Declaration of Helsinki. Ethics approval was obtained from the institutional review board of Kunming Medical University. Written informed consent was obtained from at least one parent or legal guardian of each participant.

### Anterior Chamber Depth and Ocular Biometry Measurements

Anterior chamber depth (ACD), from corneal endothelium to lens surface, was obtained using the Lenstar LS 900 (Lenstar LS 900®, Haag-Streit AG, Switzerland), a non-invasive, non-contact optical low-coherence reflectometry biometer. Other ocular biometry measurements including central corneal thickness (CCT), lens thickness (LT), axial length (AL), keratometry readings of flattest and steepest meridian (K1 and K2) were also recorded simultaneously. Refractive error was measured before and after cycloplegia using an autorefractor (RM-8000, Topcon Co., Tokyo, Japan). Supplementary Figure 1 shows the diagram of the human eye and the details of ocular biometry measurements.

### Anterior Segment Photographs Acquisition

**Figure 1:**
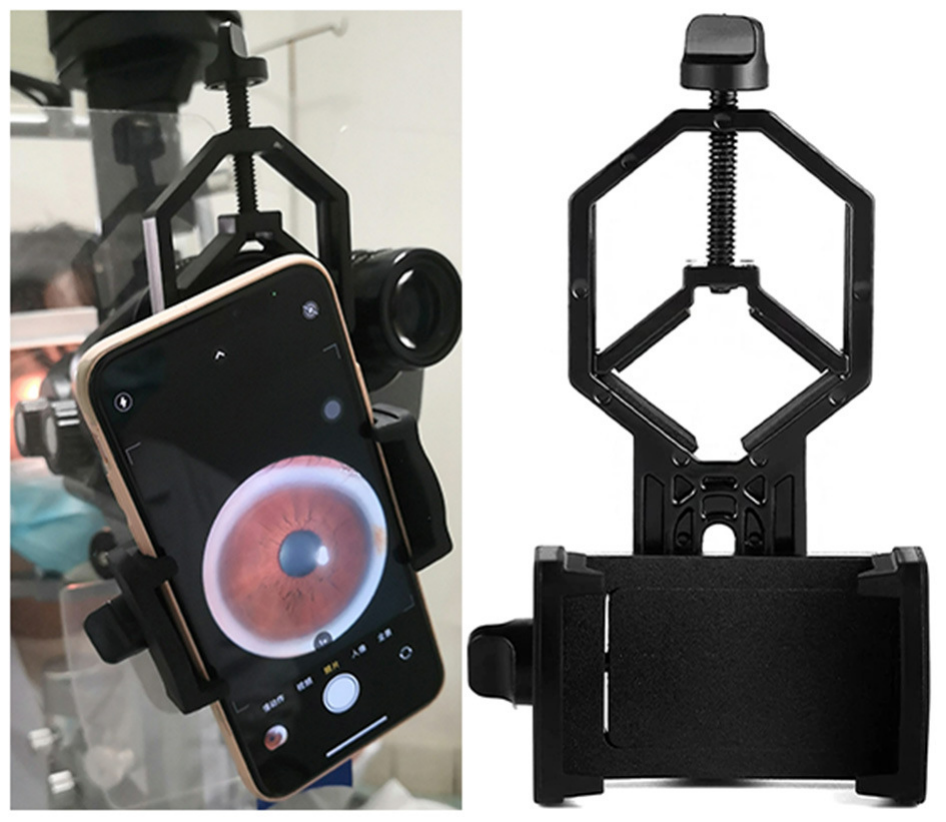
Smartphone mounted on slit lamp in use. Anterior segment photographs were captured on study eyes using a smartphone (iPhone Xs, Apple Inc, CA, USA) attached to a slit lamp. The smartphone was fixed on the eyepiece with an adapter (Celestron 81035, Celestron Acquisition LLC, CA, USA), making the camara lens in line with the eyepiece. We used the default mode of iPhone camara with a minimal magnification (1 X) to take photographs. A Bluetooth trigger for a one-tap image capture was fixed on the joystick making the procedure of taking photographs quickly and stably. Diffuse illumination of slit-lamp was used at 45-degree angle, with magnification set at 16X.

Anterior segment photographs were captured on study eyes before cycloplegia using a smartphone (iPhone Xs, Apple Inc, CA, USA) attached to a slit lamp (Figure 1). The smartphone was fixed on the eyepiece with an adapter (Celestron 81035, Celestron Acquisition LLC, CA, USA), making the camara lens in line with the eyepiece. In this study, we captured the anterior segment photographs with the light source from the slit lamp always to the left of the pupil. We used the default mode of iPhone camara with a minimal magnification (1 X) to take photographs. A Bluetooth trigger for a one-tap image capture was fixed on the joystick making the procedure of taking photographs quickly and stably. Diffuse illumination of slit-lamp was used at 45-degree angle, with magnification set at 16 X.

### Inclusion and Exclusion Criteria

The MMPS participants who had both anterior segment photographs and ACD measurements were included in this study. Participants who had pronounced opacities of the central cornea, and/or history of intraocular surgery were excluded.

### Development of the Deep Learning Algorithm

#### Neural Network Architecture

Residual Network 34 (ResNet-34) architecture was adopted in this project (18). Several modifications were introduced to ResNet-34 to finetune the model for ACD prediction. Firstly, the fully connected layer was replaced by a linear layer with an output channel of one for the regression task. No activation function was added after the linear layer. Then, the first convolutional layer was changed to one which takes in 4-channel images. Finally, the adopted ResNet-34 ended with one fully connected layer.

#### Data Preprocessing and Augmentation

Preprocessing of images was done to clean image data for model input (19). It decreases model training time and increases the model's inference speed. This process will not significantly affect the model's performance. OpenCV was used for image pre-processing in the present study. The first step for image pre-processing was cropping images to regions of interest (ROI). The original color photographs were first converted into grayscale ones and binarized using simple thresholding. Then the bounding rectangle of foreground was identified and used as ROI for the original color photographs. The images were resized to (200,200,3) after cropping, and the brightness was increased by 20%. Histogram equalization was then used to balance the RGB values of an image to enhance the contrast of images, followed by a change of color space from 3-channel to 4-channel. The last step was image normalization which scales the pixel values to zero means and unit variances. Consequently, the final input to the neural network is of size (200, 200, 4).

Image augmentation is a process to create new training examples out of the existing training data (20). This helps to adjust the current training data to generalize to other situations which allows the model to learn from a wider array of situations. To mitigate overfitting, data augmentation was used during training stage. Specifically, random rotation from −35 to 35 degrees, randomly horizontal flip with a probability of 0.5 and vertical flip with a probability of 0.1 were used.

#### Training Details and Evaluation Metrics

The dataset was randomly split into a training set and a test set with a ratio of 4:1. The batch size used is 16. Random shuffling was used for the training set. Pytorch (21), an open-source software library for DL, was used in the training and evaluation of the models. The model was trained on TITAN XP powered GPU server. Transfer learning was adopted, the ResNet-34 was loaded with a pretrained model which was trained on the ImageNet dataset which consists of 1,000 classes of objects. The modifications discussed in the architecture part were applied after loading the pre-trained weights. Adam optimizer with a learning rate of 4e-4 was used to train the model for 200 epochs (22). Mean absolute error (MAE) was used as the loss function.

#### Heat Map Generation

In order to further interpreting how the DL algorithm worked, we generated heat maps using Gradient-weighted Class Activation Mapping (Grad-CAM) algorithm (23, 24). Highlighting the important regions in hotter color, heat maps help visualization of the regions that the algorithm uses for its prediction. After normalizing the heat maps for individual images to [0, 1], we obtained the averaged heat maps across all images for an aggregated visualization.

### Statistical Analysis

The Pearson's correlation coefficient (r) was used to evaluate the correlation between predicted and measured ACD values. The MAE and coefficient of determination (*R*^2^) were used to evaluate the accuracy of prediction from the algorithm. Bland-Altman plot was used to illustrate the agreement between predicted and measured ACD values.

## Results

Of the 4,390 eyes of the MMPS 2,195 participants, we excluded 233 eyes (118 without ACD values, 115 eyes without anterior segment photographs or with poor image quality), and 4,157 eyes from 2,084 participants with both ACD values and anterior segment photographs were used to build our DL algorithm. The anterior segment photographs from these eyes were randomly distributed into a training set (3,326 photographs) and test set (831 photographs) based on a 4:1 ratio at individual level. The demographic and clinical characteristics of the eyes are presented in Table 1. The mean actual ACD in the training and test set were 3.05 ± 0.25 mm and 3.06 ± 0.25 mm, respectively.

**Table 1:** Demographic and clinical characteristics of the eyes in this study.

	**Training samples**	**Testing samples**	**Total**
Number of individuals	1,667	417	2,084
Numbers of eyes	3,326	831	4,157
Age (years)	11.6 ± 0.53	11.7 ± 0.67	11.6 ± 0.56
Gender, % Female	46%	46%	46%
Anterior chamber depth, mm	3.05 ± 0.25	3.06 ± 0.25	3.06 ± 0.26
Central corneal thickness, mm	536.41 ± 30.91	536.61 ± 33.09	536.44 ± 31.34
Lens thickness, mm	3.45 ± 0.19	3.47 ± 0.19	3.45 ± 0.19
Axial length, mm	23.48 ± 0.93	23.57 ± 1.01	23.49 ± 0.94
Keratometry readings of flattest meridian	42.79 ± 1.41	42.70 ± 1.46	42.77 ± 1.42
Keratometry readings of steepest meridian	43.88 ±1.56	43.77 ± 1.59	43.86 ± 1.57

*Data presented as mean ± SD*.

**Figure 2:**
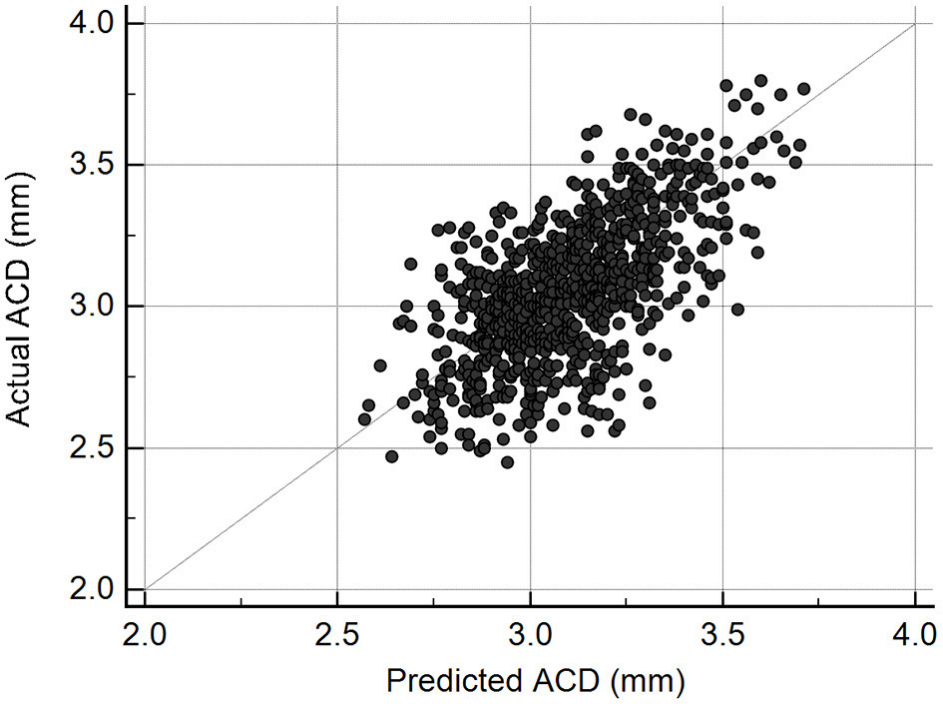
Scatterplot illustrating the relationship between deep learning-predicted and actual anterior chamber depth (ACD) measurements from Lenstar (*n* = 831, *r* = 0.63, *P* < 0.001).

The scatter plot presented in Figure 2 shows there was a good correlation (*r* = 0.63, *P* < 0.001) between ACD predictions from the DL algorithm and actual Lenstar measurements in the test set of 831 eyes. The mean difference was −0.04 ± 0.20 mm, and MAE was 0.16 ± 0.13 mm. If we set measurements less than 2.80 mm as shallow ACD (25, 26), the MAE of eyes with shallow ACD was 0.26 ± 0.16 mm (*n* = 134), and the MAE of eyes with ACD ≥ 2.80 mm was 0.14 ± 0.11 mm (*n* = 697).

**Figure 3:**
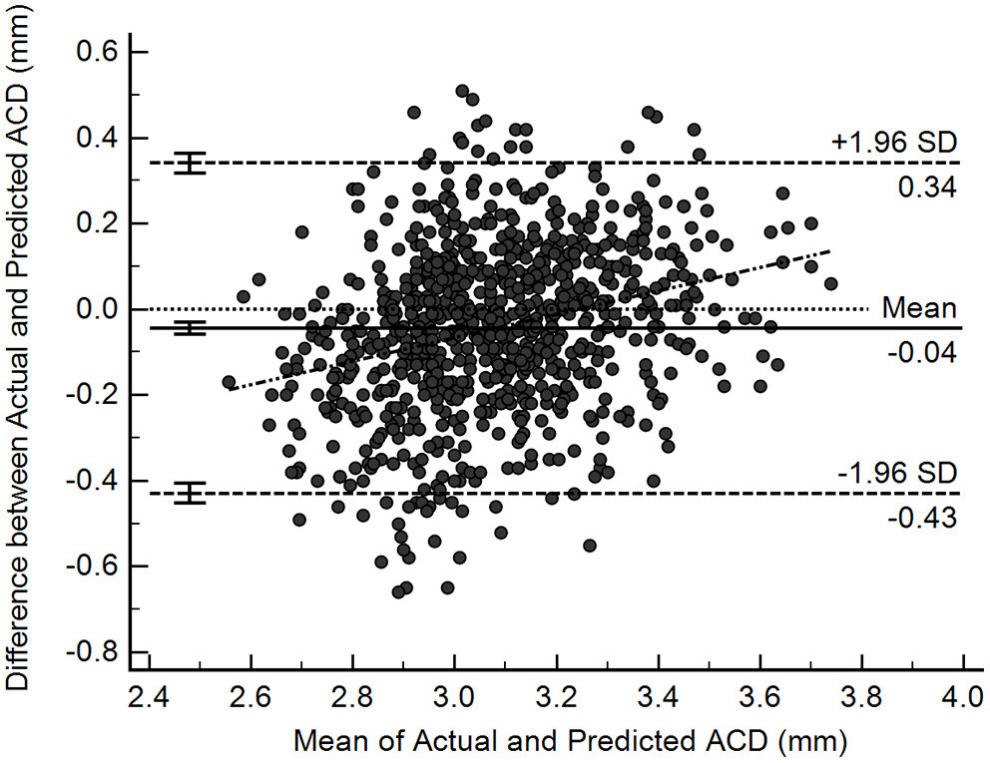
Bland-Altman plots illustrating agreement between deep learning-predicted and actual anterior chamber depth (ACD) measurements from Lenstar (*n* = 831).

Figure 3 shows the Bland-Altman plot evaluation of the agreement between predicted and measured ACD in the test samples (*n* = 831). The overall mean difference was −0.04 ± 0.20 mm, with 95% limits of agreement ranging between −0.43 and 0.34 mm. Nevertheless, there was a mild but statistically significant proportional bias (*r* = 0.27, *P* < 0.001), suggesting that at smaller range of ACD the predictions tend to give higher values than measured ACD, while at larger range of ACD, the predictions trend to give lower values than measured ACD.

**Figure 4:**
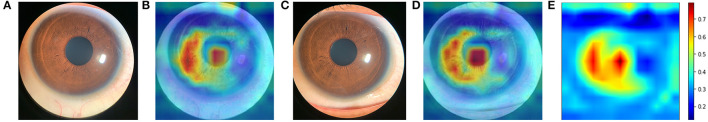
Averaged heatmap shows the regions of the anterior segment photograph that were most important for the deep learning algorithm predictions in test set. Hotter colors (reds) indicate higher activity while cooler colors (blues) represent lower activity. **(A)** an example of original anterior segment photograph (right eye) obtained by iPhone Xs; **(B)** the heatmap of the corresponding photograph; **(C)** an example of original anterior segment photograph (left eye); **(D)** the heatmap of the corresponding photograph; **(E)** averaged heat map crossed all images (*n* = 831).

Figure 4 shows examples of smartphone-obtained anterior segment photograph, the corresponding heatmap of the present neural network and the averaged heatmap crossed all images (*n* = 831). The averaged heatmap shows that the algorithm utilized regions of the central cornea in making its prediction.

## Discussion

In this study, we developed a novel DL algorithm to quantitatively predict ACD through smartphone-acquired anterior segment photographs. The predicted ACD showed good agreement with the measured ACD values. To our knowledge, this may be the first investigation to demonstrate that a DL algorithm can potentially predict the ACD through smartphone-acquired anterior segment photographs.

Our novel DL algorithm successfully predicted ACD through smartphone captured anterior segment photographs. The MAE of the predictions in test set was only 0.16 ± 0.13 mm (RMSE = 0.20 mm). The MAE of eyes with shallow ACD was bigger than the MAE of eyes with ACD ≥ 2.80 mm. That may be because of the number of eyes with ACD < 2.80 mm in training set is only 498, much less than the number of eyes with ACD ≥ 2.80 mm (*n* = 2,828). The average difference of measured and predicated ACD was −0.04 ± 0.20 mm (*P* = 0.000). However, this difference was significant statistically but not clinically as the difference was small. We captured two photographs for 50 eyes for assessing repeatability and reproducibility. For group one, the MAE of predicted ACD was 0.14 ± 0.09 mm, with 95% limits of agreement ranging between −0.36 and 0.16 mm, repeatability coefficient was 0.33 mm. For group two, the MAE was 0.14 ± 0.10 mm, with 95% limits of agreement ranging between −0.34 and 0.07 mm, repeatability coefficient was 0.33 mm. The MAE and repeatability coefficient were similar when ACD were predicted using two different photographs. Supplementary Figure 2 showed the Bland-Altman plot of the predicted ACD from group one and group two. The mean difference was −0.04 ± 0.09 mm, with 95% limits of agreement ranging between −0.20 and 0.13 mm. For the 50 eyes photographed twice, the distribution of predicted ACD was showed in Supplementary Figure 3.

A previous study that utilized machine learning to predict ACD from slit lamp images captured with a smartphone also reported a RMSE of 0.20 mm (11). However, in that study, the images used for prediction required manual maneuvering of a narrow slit (0.1 mm) which was subjective and time-consuming. In contrast, our study involved the development of a deep-learning algorithm that was trained on a much larger dataset and without manual maneuvering. Furthermore, our anterior segment photographs were captured under diffuse illumination, which suggested a two-dimensional image without slit illumination can be used to predict a third dimensional parameter, the ACD.

The overall mean difference between measured and predicted ACD in test set was −0.04 ± 0.20 mm, with 95% limits of agreement of −0.43 to 0.34 mm. Study focused on the repeatability of Lenstar showed that for ACD measurement, mean standard deviation between three consecutives measurements was 0.029, coefficient of variation was 1.06% and intraclass correlation coefficient was 0.991 (27). A previous study evaluated the agreement of ACD (ACD measurement were all from corneal epithelium to the anterior crystalline lens) measured by different instruments, including partial coherence laser interferometry (IOLMaster), scanning peripheral anterior chamber analyzer (SPAC) and anterior segment OCT (AS-OCT) (28). The 95% limits-of-agreement was: AS-OCT vs SPAC, −0.44 to 0.51 mm; AS-OCT vs. IOLMaster: −0.37 to 0.25 mm; SPAC vs. IOLMaster: −0.57 to 0.50 mm (28). Another study found that the 95% limits of agreement of ACD between Lenstar and IOL Master in eyes with cataract was −0.12 to 0.38 mm, in eyes with clear lens was −0.33 to 0.63 mm (29). The extent of agreements reported by the authors was similar to ours. Therefore, the mean difference between measured and predicted ACD is unlikely to be clinically significant. Although there was a proportional bias of our results, similar trends were observed between different methods for ACD measurement (28).

The generated saliency maps showed that the algorithm mainly utilized central corneal region in making its prediction, which was similar to another DL algorithm that predicted shallow ACD (binary classification) from Scheimpflug images (30). The hottest region was congruent with the actual measurement site of ACD which is centered on the cornea, along the visual axis from the corneal endothelium to the anterior crystalline lens capsule. Iris also played a role in making predictions. We speculate that iris was an important panel for the algorithm, like clinicians evaluate the anterior chamber in real world. The upper and right side of the iris were less used by the algorithm, that was because of the eyelid and reflex of the light make these parts less important. Randomly selected heatmaps with MAE ≤ 0.2 mm are presented in Supplementary Figure 4. We also investigated those images with poor predictions. The poor predictions were mainly attributed to dilated pupils. Randomly selected heatmaps with MAE > 0.2 mm are presented in Supplementary Figure 5. In the present study, we only excluded those participants with pronounced opacities of the central cornea, and/or with intraocular surgery history. Images with small eyelids, obscured by eyelashes, and dilated pupils were all included, to make the dataset closer to the real-word dataset, and to make the algorithm more generalizable. The MAE of the predictions with dilated pupil (*n* = 47) and un-dilated pupil (*n* = 784) was 0.22 ± 0.15 mm and 0.15 ± 0.12 mm, respectively.

Anterior chamber depth has been demonstrated to be a screening tool for angle closure glaucoma (6–8). Devereux et al. reported that, using a screening cutoff of < 2.22 mm, ACD got a sensitivity of 85% and specificity of 84% for detecting occludable angles (8). A recent study presented a higher sensitivity of 90.2% and specificity of 85.2% using the same cutoff value for distinguishing PACD from normal eyes (6). Angle closure glaucoma is an important public health problem in Asians due to its higher rate of visual morbidity. Most patients with PACG are asymptomatic, up to 64.7% of PACG cases are undetected in Asia (31). China accounts for 48% of angle closure glaucoma worldwide (2), and 90% of the cases with primary angle closure in rural China are undiagnosed (32). Gonioscopy is the current gold standard of anterior chamber angle examination. However, gonioscopy is time consuming and requires technical expertise, which limits its feasibility in large-scale population-based screening (33). ASOCT and ultrasound biomicroscopy (UBM) can help to assess the anterior chamber angle, but they are bulky, expensive and need experienced technicians. The flashlight test and van Herick's test are simple to operate. However, these two methods were reported to be of limited use as screening tests for detecting occludable angles (34).

Smartphones are increasingly used in clinical settings to provide high quality images (35, 36). Coupled with DL algorithms, smartphones may be used for detecting ocular diseases. For example, smartphone based anterior segment photographs and retinal images for cataract grading, glaucoma and diabetic retinopathy detection have been reported (12, 36, 37). There are plenty of advantages for smartphones used in clinics. Since smartphones are widely available, they provide a low-cost and universally accessible method to capture high resolution ocular images. Smartphones usually have a large data storage capacity and do not require extra computers for image storage or processing. In addition, the images captured by smartphones can be easily transmitted wirelessly for consultation in real time. These advantages make smartphone a useful tool in clinics and can bring great benefits for tele-consultation or screenings in remote areas. A previous study successfully developed a machine learning system using anterior segment images captured by digital camera under visible wavelength to diagnose anterior segment eye abnormalities (38). It is conceivable that eye images captured under nature light by smartphone without extra equipment could provide many useful information for ophthalmologist with the help of artificial intelligence. As such it may be used by a wide potential audience and locations, especially in rural area and developing countries.

There are several strengths in the present proof-of-concept study. First, this may be the first study to use DL to quantitatively predict ACD through smartphone-acquired anterior segment photographs. The generated saliency maps showed that the algorithm mainly utilized central corneal region in making its prediction, which was congruent with the actual measurement site of ACD. Secondly, by using merely a smartphone we obtained high quality of anterior segment photographs. Simple instrument makes more cost effective and sustainable. These images were captured under diffuse illumination without slit beam, which makes the procedure much easier and reproduceable.

There are also some limitations in our study. Participants were all from a school-based cohort study aged 11–15 years old, and there were no PACD patient included. Hence, further training of the algorithm involving eyes of older participants, and PACD eyes are needed. Nevertheless, the present study is a proof-of-concept study, which demonstrated that smartphone-acquired anterior segment images can potentially be used to estimate ACD *via* DL.

## Conclusion

In conclusion, we developed a novel method to estimate ACD using DL algorithm based on smartphone-acquired anterior segment photographs. Further refinement and training involving older participants PACD eyes are still needed, followed up further external validations. This is particularly important in China where the risk of PACG is high and often undetected, leading to increased risk of vision impairment.

## Data Availability Statement

The raw data supporting the conclusions of this article will be made available upon further inquiries to the corresponding author.

## Ethics Statement

The studies involving human participants were reviewed and approved by Kunming Medical University. Written informed consent to participate in this study was provided by the participants' legal guardian/next of kin.

## Author Contributions

All authors listed have made a substantial, direct, and intellectual contribution to the work and approved it for publication.

## Conflict of Interest

The authors declare that the research was conducted in the absence of any commercial or financial relationships that could be construed as a potential conflict of interest.

## Publisher's Note

All claims expressed in this article are solely those of the authors and do not necessarily represent those of their affiliated organizations, or those of the publisher, the editors and the reviewers. Any product that may be evaluated in this article, or claim that may be made by its manufacturer, is not guaranteed or endorsed by the publisher.

## Supplementary Material

Supplementary Figure 1 Diagram of the human eye. CCT, central corneal thickness; ACD, anterior chamber depth (back of cornea to front of lens); LT, lens thickness; AL, axial length.

Supplementary Figure 2 Bland-Altman plots illustrating agreement between the predicted ACD from one eye using two anterior segment photographs (*n* = 50).

Supplementary Figure 3 Overlapping histogram of the measurements. For the 50 eyes photographed twice, the distribution of predicted anterior chamber depth overlapped well.

Supplementary Figure 4 Randomly selected heatmaps with MAE ≤ 0.2 mm. The regions of the central corneal were most important for the deep learning algorithm making predictions. RE, Right eye; LE, Left eye; GT, Ground truth; Pred, prediction (mm).

Supplementary Figure 5 Randomly selected heatmaps with MAE > 0.2 mm. The hottest regions were not the central corneal. Most of them had mid-dilated pupils. RE, Right eye; LE, Left eye; GT, Ground truth; Pred, prediction (mm).
