# Supplementary figures and images for: Smartphone-Acquired Anterior Segment Images for Deep Learning Prediction of Anterior Chamber Depth: A Proof-of-Concept Study
Source: Front Med (Lausanne). 2022 Jun 23;9:912214. doi: 10.3389/fmed.2022.912214 (PMC9259953; doi:10.3389/fmed.2022.912214)

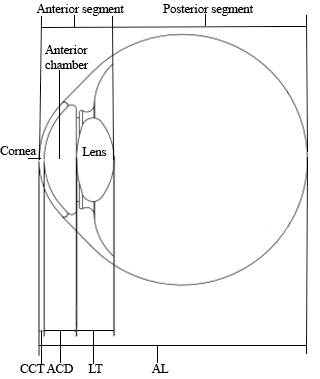

Supplement: Supplementary Figure 1 — Diagram of the human eye. CCT, central corneal thickness; ACD, anterior chamber depth (back of cornea to front of lens); LT, lens thickness; AL, axial length. [file Image_1.TIF]

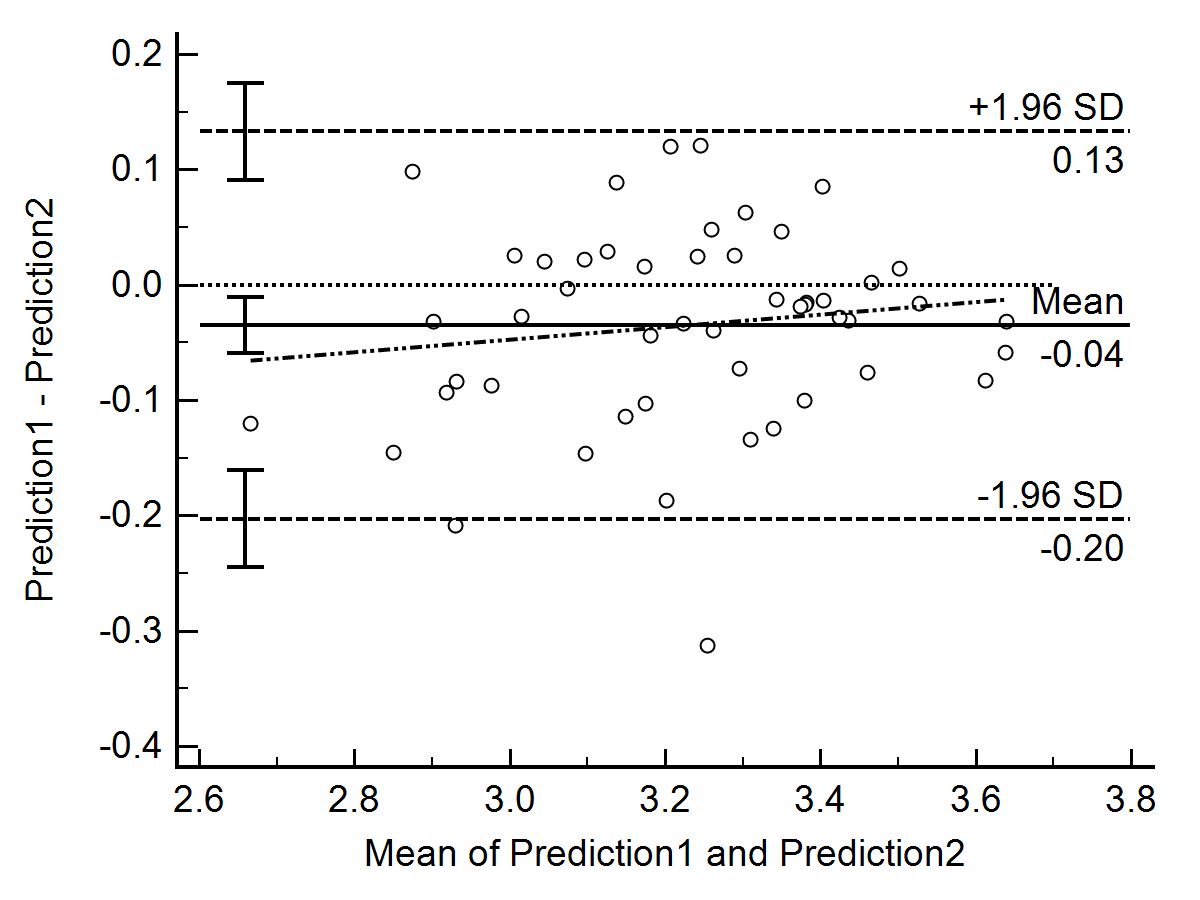

Supplement: Supplementary Figure 2 — Bland-Altman plots illustrating agreement between the predicted ACD from one eye using two anterior segment photographs (n = 50). [file Image_2.TIF]

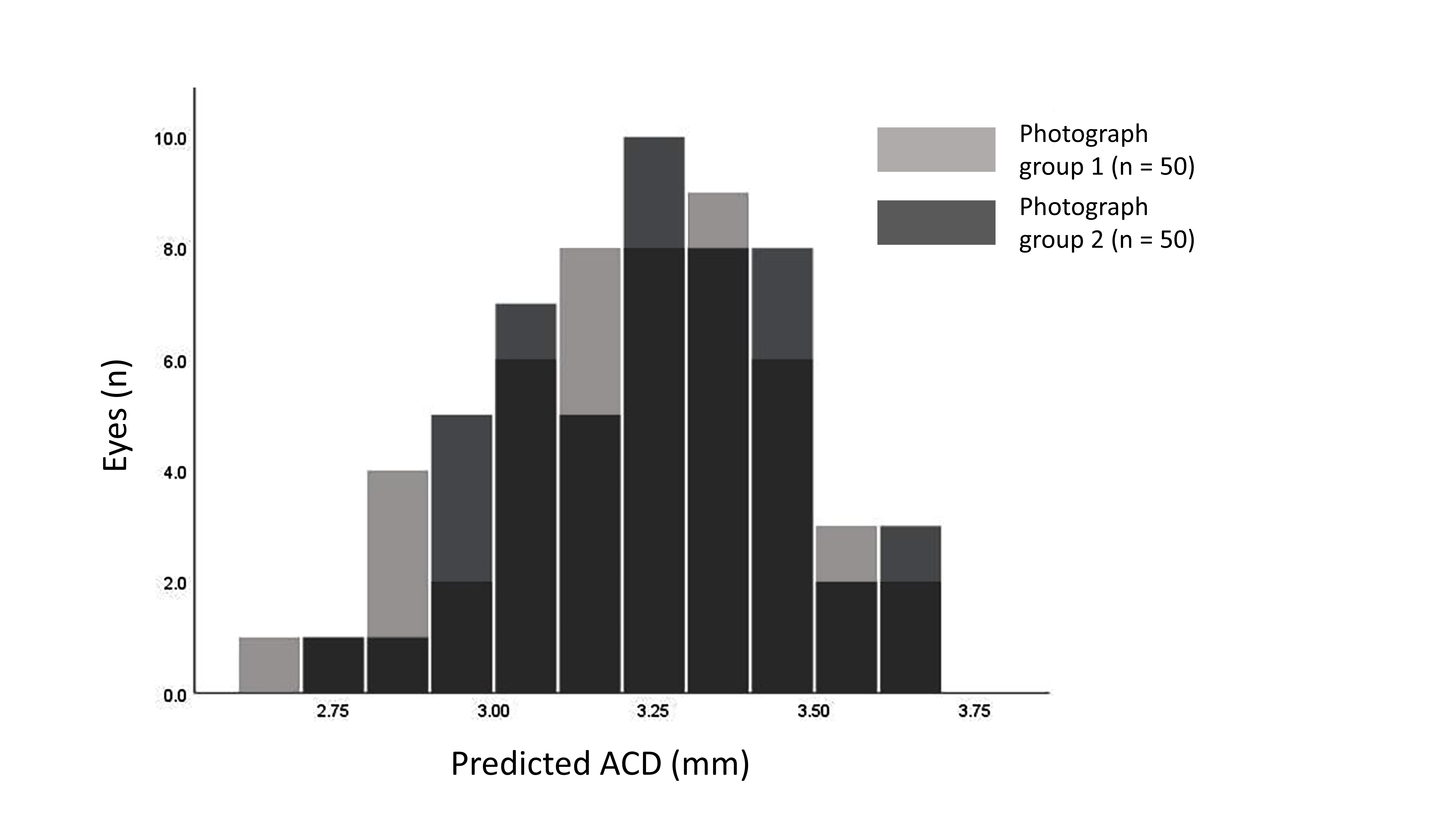

Supplement: Supplementary Figure 3 — Overlapping histogram of the measurements. For the 50 eyes photographed twice, the distribution of predicted anterior chamber depth overlapped well. [file Image_3.TIF]

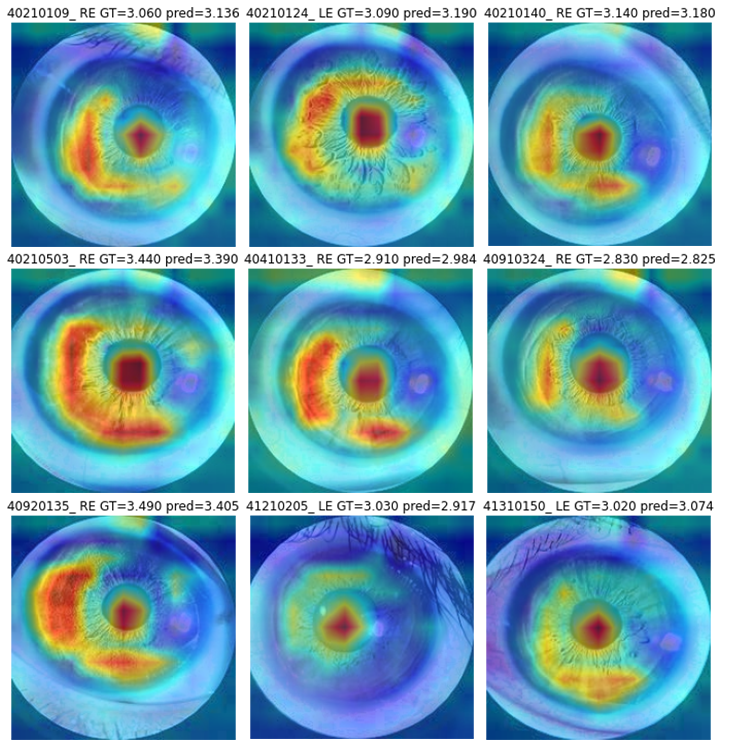

Supplement: Supplementary Figure 4 — Randomly selected heatmaps with MAE ≤ 0.2 mm. The regions of the central corneal were most important for the deep learning algorithm making predictions. RE, Right eye; LE, Left eye; GT, Ground truth; Pred, prediction (mm). [file Image_4.TIF]

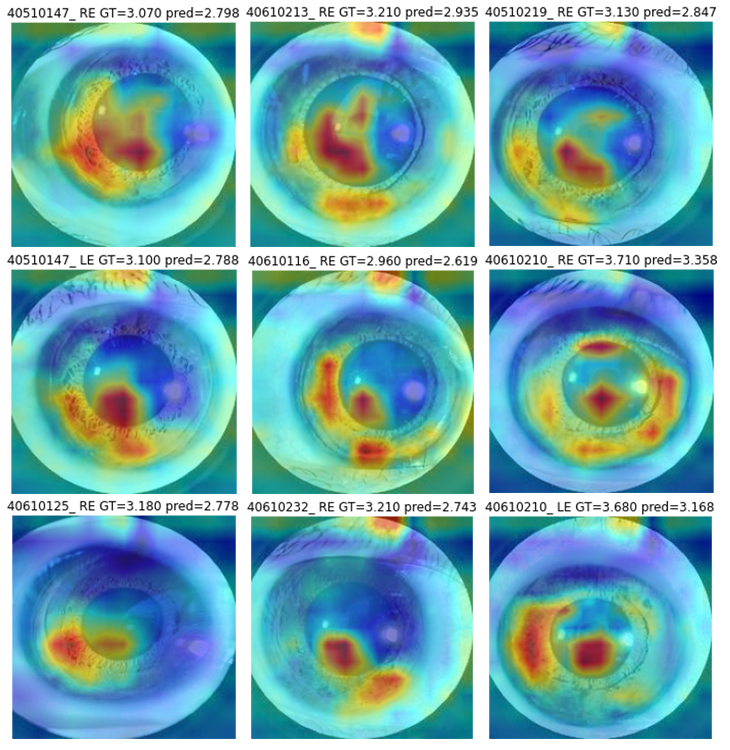

Supplement: Supplementary Figure 5 — Randomly selected heatmaps with MAE > 0.2 mm. The hottest regions were not the central corneal. Most of them had mid-dilated pupils. RE, Right eye; LE, Left eye; GT, Ground truth; Pred, prediction (mm). [file Image_5.TIF]
